# Microbial determinants of soil quality in mixed larch and birch forests: network structure and keystone taxa abundances

**DOI:** 10.3389/fpls.2025.1491038

**Published:** 2025-07-15

**Authors:** Zhaoxuan Ge, Xinyu Zhang, Chang Liu, Minghao Li, Ruihan Wang, Yang Zhang, Zhidong Zhang

**Affiliations:** College of Forestry, Hebei Agricultural University, Baoding, China

**Keywords:** *Larix principis-rupprechtii*, microbial diversity, network complexity, network stability, keystone taxa

## Abstract

Changes in forest soil microbial community characteristics affect soil function and quality. However, the mechanisms through which microbes drive soil quality across different stand types remain unclear. Three typical forest types, larch (*Larix principis-rupprechtii*) forest (LF), birch (*Betula platyphylla*) forest (BF), and mixed larch and birch forest (MF), were selected to assess soil properties, microbial community characteristics, and the complexity and stability of co-occurrence networks. The results showed that stand type significantly affected soil quality, microbial community composition, and network structure. Compared to LF stands, both MF and BF stands exhibited higher levels of soil organic carbon (SOC), total nitrogen (TN), available nitrogen (AN), available phosphorus (AP), maximum water holding capacity (MWHC), and soil quality index (SQI), with the SQI increasing by 54.29% and 48.57%, respectively. The bacterial Shannon index was lower in MF and BF stands, whereas the fungal Shannon index was higher. Fungal community composition was more sensitive to variations among the three stand types than bacterial communities. The MF stands exhibited higher microbial complexity and stability, with a higher relative abundance of keystone bacterial and fungal taxa associated with nutrient cycling and transformation. These findings suggest that SQI can be enhanced by increasing soil fungal diversity, improving microbial network complexity and stability, and increasing the relative abundance of key microbial taxa. This study emphasized that the mixing of larch and birch significantly affected soil microbial community characteristics, which in turn impacted soil nutrient utilization. The insights gained provide a deeper understanding of soil nutrient cycling in plantation ecosystems, offering valuable references for sustainable forest management practices.

## 1 Introduction

Soil microorganisms play a vital role in soil nutrient cycling and ecosystem function, and have received extensive attention (Falkowski et al., 2008; Lu et al., 2023). It is widely recognized that tree species characteristics strongly influence soil microbial community composition and function through both direct and indirect pathways (Shao et al., 2017; Otsing et al., 2021). Direct influences include the release of root exudates and the deposition of aboveground litter. The quantity and composition of root exudates—such as organic acids and sugars—vary among tree species, providing specific substrates and nutrients that selectively support distinct microbial taxa. Similarly, the chemical properties of litterfall directly influence microbial decomposition rates and community structure (Prescott and Grayston, 2013; Bai et al., 2023). For example, Garau et al. (2019) reported that litter in broad-leaved forests generally contains higher nitrogen and phosphorus concentrations and supports more microorganisms that use soil carbon sources compared to coniferous litter. Indirectly, tree species can modify the soil microenvironment by altering pH, moisture content, and nutrient availability, thereby creating conditions that favor specific microbial assemblages (Khlifa et al., 2017; Chen et al., 2019b). For instance, Li et al. (2023) found that tree species-mediated differences in stand types significantly affected soil nutrient concentrations, which in turn served as the primary driver of changes in soil bacterial and fungal diversity. Moreover, Jiang et al. (2021) demonstrated fungal communities are more sensitive to environmental changes compared to bacterial communities. Therefore, exploring the relationship between tree species and soil microbes is crucial for understanding soil nutrient cycling processes and holds significant implications for effective forest management.

Soil microbial keystone taxa play a key role in maintaining ecological networks and functions that enable the efficient use of resources (Banerjee et al., 2018; Hernandez et al., 2021). Co-occurrence network analysis has become a widely used tool to identify keystone taxa (Berry and Widder, 2014). Furthermore, analyzing multiple topological features of microbial networks (e.g., indices of edges, nodes, and mean degree) can reveal the complexity and stability of microbial communities (Goberna and Verdu, 2022). This approach aids in understanding how microbial network structure responds to environmental changes (Collyer et al., 2023). Growing evidence suggests that both network structure and keystone taxa have important implications for ecosystem multifunctionality (Qiu et al., 2021). For instance, Huo et al. (2023) revealed that pine-oak mixed forest altered the structure of fungal community networks. Conversely, He et al. (2022) found that the co-occurrence network of soil microbial communities became more complex in pure forests dominated by *Phoebe bournei*. Li et al. (2023) reported that *symbiotrophic* fungi were more abundant in coniferous and broad-leaved mixed forests, whereas *saprophytic* fungi were more prevalent in coniferous pure forest. Despite these insights, the effects of changes in tree species characteristics on microbial networks and keystone taxa remain poorly understood.

Soil quality represents the fertility, availability, and restorability of soil, reflecting its overall ecological function (Bunemann et al., 2018). It is essential for sustaining biological productivity and promoting the health of plants and animals (Schloter et al., 2003). Various methods for evaluating soil quality have been proposed, with the soil quality index (SQI) currently being widely applied (Gonzaga et al., 2016; Cui et al., 2024). The SQI method usually groups soil physicochemical and biochemical properties into a minimum dataset, integrating them into a single numerical value through a scoring equation for comprehensive evaluation (Bastida et al., 2008; Qiu et al., 2019; Li et al., 2024). Therefore, soil quality assessed through the SQI enhances our understanding and prediction of soil ecological functions and its capacity to respond to environmental change. Additionally, soil microorganisms are closely related to soil ecosystems and can further influence soil quality (Delgado-Baquerizo et al., 2020; Liu et al., 2024). Wan et al. (2024) noted that changes in fungal community composition and reductions in bacterial diversity improved SQI on the Loess Plateau. Liu et al. (2024) revealed that microbial diversity, particularly the Shannon index ratio of fungi to bacteria and the relative abundance of specific dominant taxa such as *Ascomycota* and *Acidobacteriota* have been identified as key drivers of soil quality. However, current research primarily focuses on agricultural soils and methods for evaluating the soil quality index, with few studies exploring how soil microorganisms affect soil quality across different stand types.

Larch (*Larix principis-rupprechtii*), a widely used afforestation species, grows well in the subalpine regions of northern China, offering advantages such as fast growth, high-quality wood, and effective soil and wind retention, and high stress resistance (Ge et al., 2023). However, the rapid expansion of pure larch forests has led to issues such as low forest productivity and stand instability, which pose significant threats to the multifunctional benefits of these forests (Cheng et al., 2023). Birch (*Betula platyphylla*) is commonly used as a mixed tree species in larch forests of North China. Mixed-species stands tend to support more diverse community structures and influence soil nutrient conditions and microbial communities (Zhang and Chen, 2007; Gillespie et al., 2021). However, their effects on soil quality remain unclear. In this study, we collected soil samples from larch forests, birch forests, and larch-birch mixed forests to examine variations in soil properties and microbial communities across different stand types. The goals were to explore the effects of microbial community characteristics on soil quality and to identify the underlying mechanisms. Specifically, this study aimed to address the following questions: (1) What are the differences in soil physicochemical properties and SQI among the stand types? (2) How do microbial community characteristics (diversity, composition, microbial network, and keystone microbial taxa) differ among stand types? (3) What are the specific mechanisms through which microbial community characteristics (diversity, composition, microbial network, and keystone taxa) influence soil quality?

## 2 Materials and methods

### 2.1 Study site

**Figure 1:**
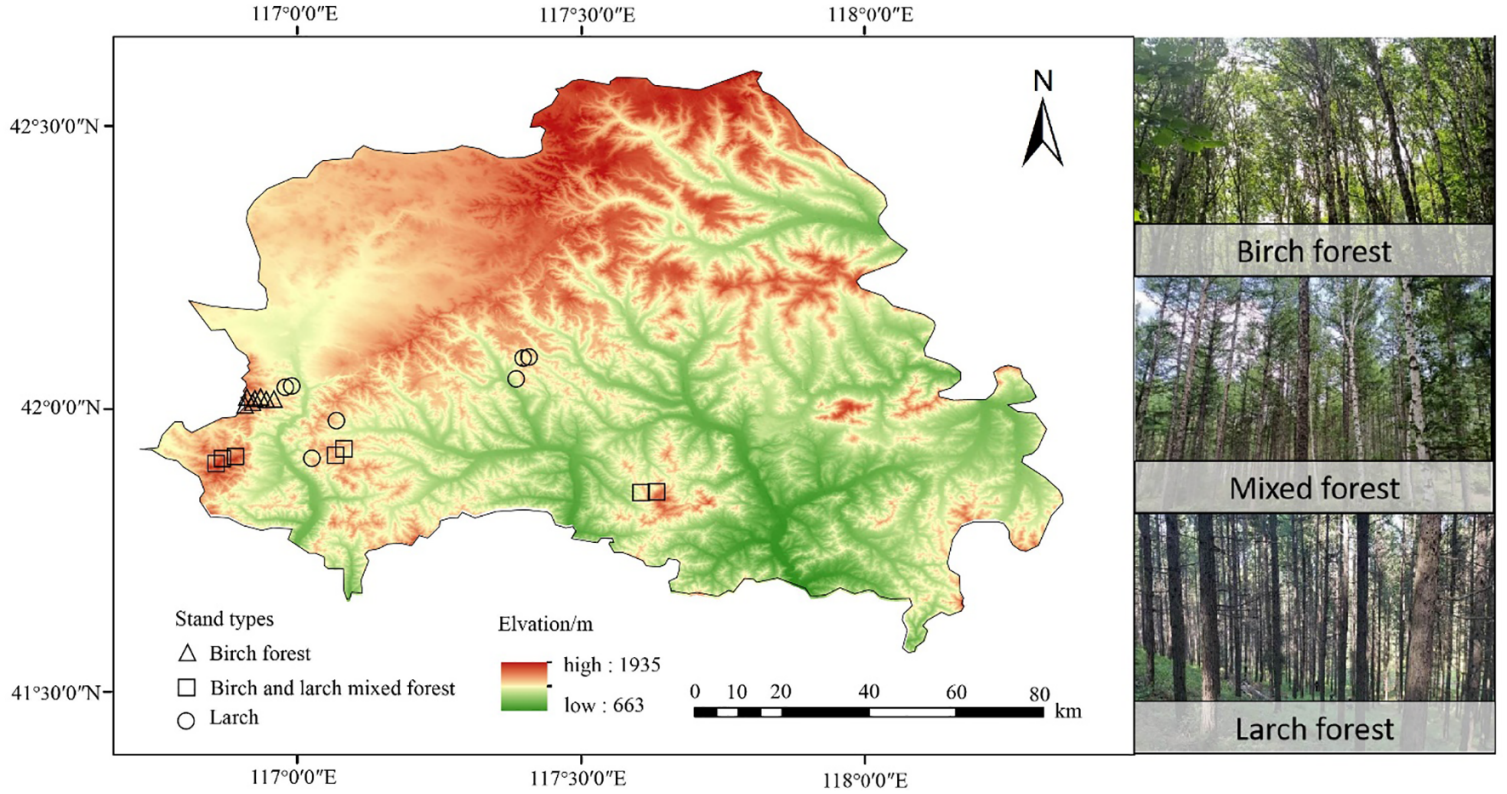
Study area and sample plots distribution in Weichang County, Hebei Province, Northern China.

The study area is located at the forest region in Weichang County, Hebei Province, China (from 41°35′ N to 42°40′N, 116°32′E to 117°14′E) (**Figure 1**). The elevation ranges from 663 to1935 m. This region has a typical cold-temperate continental monsoon climate, with a mean annual temperature ranging from -1.4 to 4.7°C and a mean annual precipitation of 380 to 560 mm. The main tree species include larch, birch, *Picea asperata*, and *Populus davidiana.*

### 2.2 Experiment design

Three typical stand types were selected in the region at altitudes of 1300–1600 m, all with similar slopes and aspects: larch forest (LF), birch forest (BF), and mixed larch and birch forest (MF). The birch forests are natural secondary forests, while the mixed forests typically result from the artificial replanting of larch in the understory of BF. The larch forests studied in this study are first-generation plantations established on land that had experienced severe ecological degradation. Prior to the Qing Dynasty, the region was primarily covered by natural forests and grasslands. However, extensive human disturbances—such as overlogging, intensive grazing, and frequent wildfires—led to significant ecosystem deterioration. By the mid-20th century, these activities had nearly eradicated the native forests. In response, systematic restoration efforts were initiated, with large-scale afforestation projects implemented using larch as the dominant species. Tree ring analysis indicated that the trees are approximately 40–50 years old. For each stand type, seven plots (30 × 30 m) were established, yielding a total of 21 plots (**Figure 1**). All standing trees with a diameter at breast height (DBH) ≥ 5 cm were numbered, and their species name, DBH, height, and other relevant information were measured and recorded. The details of the sample plots are presented in **Table 1**.

**Table 1:** Basic stand information of the sampling plots.

Stand types	Tree species	Altitude (m)	Stand age (years)	DBH (cm)	Height (m)	Stand density (trees·ha^-1^)
LF	*Larix principis-rupprechtii*	1333 ± 82	40-50	16.52 ± 2.75	14.39 ± 2.18	1343 ± 210
MF	*L*. *principis-rupprechtii* *Betula platyphylla*	1489 ± 96	50-60	21.21 ± 2.36	16.64 ± 3.51	1073 ± 479
BF	*B*. *platyphylla*	1501 ± 87	40-55	18.94 ± 2.49	15.46 ± 1.27	1294 ± 352

Data are means ± SE. LF, larch forest, MF, mixed larch and birch forest, BF, birch forest. DBH: diameter at breast height.

### 2.3 Data collection

#### 2.3.1 Sample collection and soil properties measurements

In August 2022, soil samples were collected from each sample plot using the five-point sampling method. After removing the top layer of litter, a soil auger with an inner diameter of 4 cm was used to extract soil from a depth of 0–15 cm. The samples were mixed to form a single composite sample, which was then divided equally into two parts. A total of 42 soil samples were collected (2 samples per plot, 21 plots in total). One portion of the soil samples was filtered through a sieve to remove plant roots and stones, placed into 20 ml centrifuge tubes, and stored at -80°C for subsequent high-throughput sequencing of microorganisms. The other portion was placed on a cool lab bench to dry naturally for the determination of soil physicochemical properties. Soil bulk density (BD), maximum water holding capacity (MWHC), pH, soil organic carbon (SOC), total nitrogen (TN), available nitrogen (AN), total phosphorus (TP), and available phosphorus (AP) were measured using standard soil agrochemical analysis methods (Bao, 2000). Specifically, BD was measured using 100 cm³ stainless steel corers, oven-drying at 105°C to constant mass. MWHC was determined by 24-hour saturation of undisturbed cores (100 cm³) with free water removal. Soil pH was measured using pH meter in a soil: water (1:2.5) extract. SOC was quantified via potassium dichromate oxidation with external heating. TN was measured using the Kjeldahl digestion method. TP was evaluated via the molybdenum-antimony colorimetry. AN was measured using the Alkaline hydrolysis diffusion method, AP was using Olsen’s bicarbonate extraction method.

#### 2.3.2 Soil DNA extraction, sequencing, and data processing

Nucleic acids were extracted from 0.2 and 0.5 g of soil samples using the OMEGA Soil DNA Kit (D5635-02) (Omega Bio-Tek, Norcross, GA, USA). The extracted DNA was electrophoresed on a 0.8% agarose gel to determine molecular size and quantified using a Nanodrop. For the soil bacteria project, the highly variable V3-V4 region of the bacterial rRNA 16S gene, approximately 468 bp in length, was chosen for sequencing (Callahan et al., 2016). PCR amplification was performed using the primers 338F (5′-barcode + ACTCCTACGGGAGGCAGCA-3′) and 806R (5′-GGACTACHVGGGTWTCTAAT-3′), targeting the 16S rRNA V3-V4 region of bacteria. For the soil fungal project, the ITS1 fungal region was amplified and sequenced using primers ITS5 (GGAAGTAAAAGTCGTAACAAGG) and ITS2 (GCTGCGTTCTTCATCGATGC), with a length of about 280 bp. The PCR reaction components were set up, and template DNA was pre-denatured at 98 °C for 5 minutes to ensure complete denaturation, followed by the amplification cycle. PCR products were quantified using a Microplate reader (BioTek, FLx800) with the Quant-iT PicoGreen dsDNA Assay Kit. Quantified products for each sample were mixed based on the desired data amount and used for library preparation with Illumina’s TruSeq Nano DNA LT Library Prep Kit. Microbiome data were analyzed using QIIME2 version 2019.4, following a modified and refined process based on the official tutorial (https://docs.qiime2.org/2019.4/tutorials/, accessed on 14 June 2023) (Bolyen et al., 2019). High throughput sequencing was carried out on the Gene Cloud platform supplied by Shanghai Paisano Biotechnology Co. Alpha diversity indices including the Shannon index, and Chao1 index were calculated using QIIME2 (2019.4) to evaluate the diversity of the microbiota within the samples.

#### 2.3.3 Calculation of soil quality index

The calculation of the SQI typically involves three steps (Huang et al., 2018): 1) selecting the minimum data set (MDS) that best represents soil function using principal component analysis (PCA) (**Supplementary Table S1**), 2) scoring the selected indicators (**Supplementary Table S2**), and 3) calculating the SQI using the weighted index method (**Supplementary Table S2**). Eight soil physicochemical properties were analyzed, and PCA was employed to identify the most appropriate soil quality indicators. Principal components (PCs) with eigenvalues > 1.0 were considered for indicator selection (Andrews et al., 2002). The MDS retained only the most highly weighted indicators, specifically those within the top 10% of the highest weight loadings. When multiple indicators were retained in each PC, Pearson correlation analysis was used for further screening (**Supplementary Table S3**). If the high-weight indicators were uncorrelated (correlation coefficient< 0.7), each indicator was included in the MDS. Otherwise, the indicator with the highest weight value was retained (Yu et al., 2018).

After selecting the MDS metrics, a nonlinear scoring function was used to convert the soil metrics into scores ranging from 0 to 1. The calculations were based on Equation 1


(1) $$SNL=\frac{1}{1+{\left(\frac{x}{x_{0}}\right)}^{b}}$$

where SNL represents the nonlinear score for each indicator, taking values in the range 0-1. x is the indicator value, $x_{0}$ is the average of the corresponding indicators, and b is a shape parameter determining the steepness of the non-linear scoring function. The parameter b is set to -2.5 for indicators where “the more, the better” applies, and 2.5 for indicators where “the less, the better” applies (Raiesi, 2017).

After scoring and weighting all MDS indicators, The SQI was calculated using Equation 2 (Masto et al., 2008):


(2) $$SQI=\sum_{i=1}^{n}\left(W_{i}\times SNL_{i}\right)$$

where $W_{i}$ is the weight of the indicator, calculated as the ratio of the variance of the common factor of the indicator to the total variance in the PCA. n is the number of indicators in the MDS.

#### 2.3.4 Co-occurrence network and keystone taxa

Microbial molecular networks were constructed to determine the effects of stand types on soil microbiomes and identify potential keystone taxa. To minimize spurious correlations, bacterial and fungal amplicon sequence variant (ASV) occurrences were required to exceed 1/5 of the sample size, and their relative abundance had to be greater than 0.01% (Faust, 2021). Spearman correlation analyses were performed using ASV abundance tables for bacteria and fungi, and correlation coefficients (r) and *p*-values were obtained (Barberan et al., 2014), with *p*-values adjusted for multiple comparisons using Benjamini-Hochberg false discovery rate (FDR) method, applying a significance threshold of 0.05. Correlations with | r | > 0.7 and *p<* 0.05 were considered statistically significant and used to construct the networks. Co-occurrence network analyses were conducted using the ‘Hmisc’ R package (Harrell, 2024). Network topological parameters, including the number of nodes, number of edges, mean degree, density, network diameter, and mean path length, were extracted using the ‘igraph’ package (Csárdi et al., 2024). Network visualization was performed using Gephi 0.10.1 (https://gephi.org/). Subsequently, the subgraph function in the ‘igraph’ package was used to extract the sub-network for each sample. The average value of the sub-network’s topological parameters was standardized to directly assess the impact of microbial network complexity on SQI. Network stability was characterized by the remaining proportion of nodes and robustness. In this study, robustness was defined as the proportion of remaining species in the network after 50% node removal (Montesinos-Navarro et al., 2017). The topological role of each node was calculated based on its within-module connectivity ( $Z_{i}$ ) and among-module connectivity ( $P_{i}$ ) (Guimerà and Nunes Amaral, 2005). According to previous research, module hubs ( $Z_{i}\geq 2.5$ , $P_{i}<0.62$ ), connectors ( $Z_{i}<2.5$ , $P_{i}\geq 0.62$ ), and network hubs ( $Z_{i}\geq 2.5$ , $P_{i}\geq 0.62$ ) were defined as keystone taxa (Banerjee et al., 2019), as illustrated in **Supplementary Figure S1**.

### 2.4 Data analysis

The main effects of stand type on soil physicochemical properties, SQI, and microbial alpha diversity were examined using one-way analysis of variance (ANOVA), following tests for normality and homogeneity of variance. Multiple comparisons among different stand types were performed using Tukey’s HSD test (*P*< 0.05). Microbial beta diversity was assessed by Principal Coordinate Analysis (PCoA) based on the Bray-Curtis distance matrix, using the R package “vegan”. The analysis of similarity (ANOSIM) was conducted with the “anosim” function in R to compare the similarity in soil microbial community structure. Potential ecological functions were predicted using FAPROTAX and FUNGuid. Bacterial and fungal diversity were quantified by z-transforming the Chao 1 and Shannon indices, respectively. These diversity indices were then regressed to explore variation in SQI in relation to bacterial and fungal diversity, microbial network complexity, and the abundance of keystone taxa. All data analyses were conducted using R 4.3.2 software (R Core Team, 2023).

## 3 Results

### 3.1 Soil properties and SQI in different stand types

The results indicated that all metrics varied significantly among the stand types (*P<* 0.05) (**Table 2**). The contents of SOC, TN, AN, AP, and MWHC in MF were similar to that in BF but higher than in LF. No significant difference was observed in the TP content between LF and BF, although it was significantly lower in MF. BD was higher in LF, while pH was higher in BF. Additionally, stand type had a significant effect on SQI (*P*< 0.05). MF exhibited the highest soil quality, which did not differ significantly from that of BF, whereas LF had the lowest soil quality.

**Table 2:** Indicators of soil physical and chemical properties in three stand types.

Indicators	LF	MF	BF
SOC	63.19 ± 12.57^a^	121.01 ± 30.49^b^	100.47 ± 6.71^b^
TN	3.08 ± 0.52^a^	5.46 ± 1.21^b^	4.55 ± 0.63^b^
TP	0.43 ± 0.21^a^	0.97 ± 0.25^b^	0.64 ± 0.15^a^
AN	293.16 ± 49.89^a^	549.19 ± 122.25^b^	505.98 ± 73.18^b^
AP	8.00 ± 2.5^a^	12.93 ± 1.8^b^	13.39 ± 1.7^b^
pH	6.19 ± 0.25^ab^	6.06 ± 0.09^a^	6.35 ± 0.12^b^
MWHC	37.87 ± 3.26^a^	44.41 ± 2.36^b^	44.12 ± 4.58^b^
BD	0.97 ± 0.09^a^	0.84 ± 0.1^ab^	0.84 ± 0.09^b^
SQI	0.35 ± 0.01^a^	0.54 ± 0.05^b^	0.52 ± 0.02^b^

SOC, soil organic carbon concentration (g kg^-1^); TN, total nitrogen content (g kg^-1^); TP, total phosphorus content (g kg^-1^); AN, available nitrogen content (g kg^-1^); AP, available phosphorus content (g kg^-1^); MWHC, maximum water holding capacity; BD, bulk density. Values are means ± standard deviation (n = 7). Different lowercase letters indicate significant differences among stand types at *P*< 0.05 according to Tukey’s HSD test.

### 3.2 Change in soil microbial community diversity

**Figure 2:**
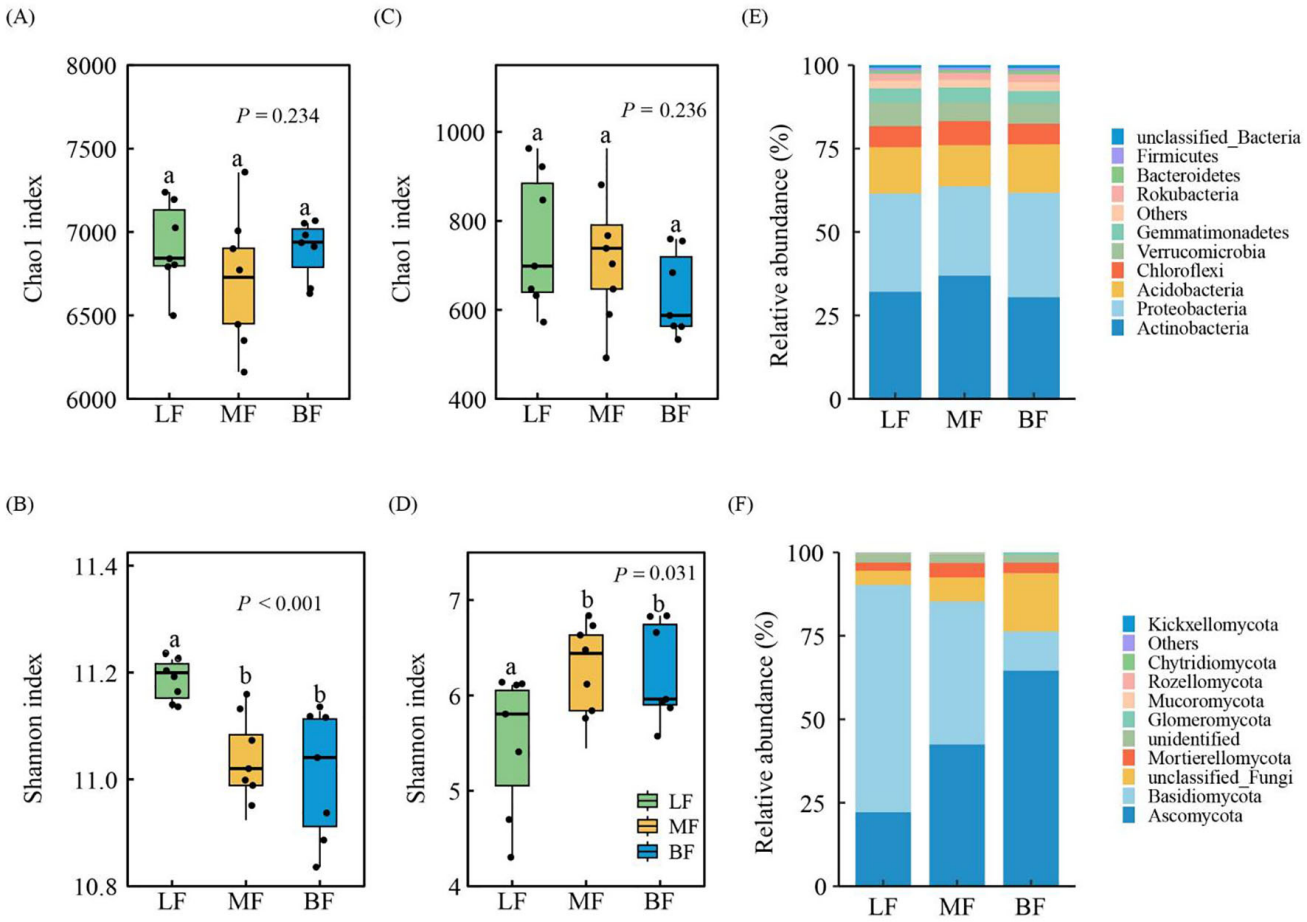
Alpha diversity indices for soil bacterial **(A, B)** and fungal **(C, D)** communities, and taxonomic composition of soil bacterial **(E)** and fungal **(F)** communities at the phylum level. Different lowercase letters indicate significant differences among stand types at *P<* 0.05.

The Chao1 richness index for soil bacteria and fungi showed no significant differences among the stand types (*P* > 0.05, **Figures 2A, C**). However, the Shannon index diversity for bacteria and fungi in MF and BF was similar, with significant differences observed when compared to LF (*P<* 0.05, **Figures 2B, D**). Specifically, the bacterial Shannon index was lower in MF and BF, while the fungal Shannon index was higher. The abundance distribution of bacterial and fungal phyla varied among the stand type. The dominant bacterial phyla were Actinobacteria, Proteobacteria, and Acidobacteria, collectively accounting for approximately 75% of the total bacterial population (**Figure 2E**). The most abundant fungal phyla were Basidiomycota, followed by Ascomycota, which together accounted for over 75% of the total fungal population (**Figure 2F**). The five most abundant bacterial taxa were *norank*_*c*_*Subgroup*_*6*, *Candidatus Udaeobacter*, *Xanthobacteraceae*, *norank*_*c*_*KD4-96*, and *Mycobacterium*, which collectively comprised 23.74% of the bacterial community. Similarly, the five most abundant fungal genera were *Sebacina*, *Russula*, *Exophiala*, *Mortierella*, and *Inocybe*, which comprised 27.54% of the fungal community, on average (**Supplementary Figure S2**).

**Figure 3:**
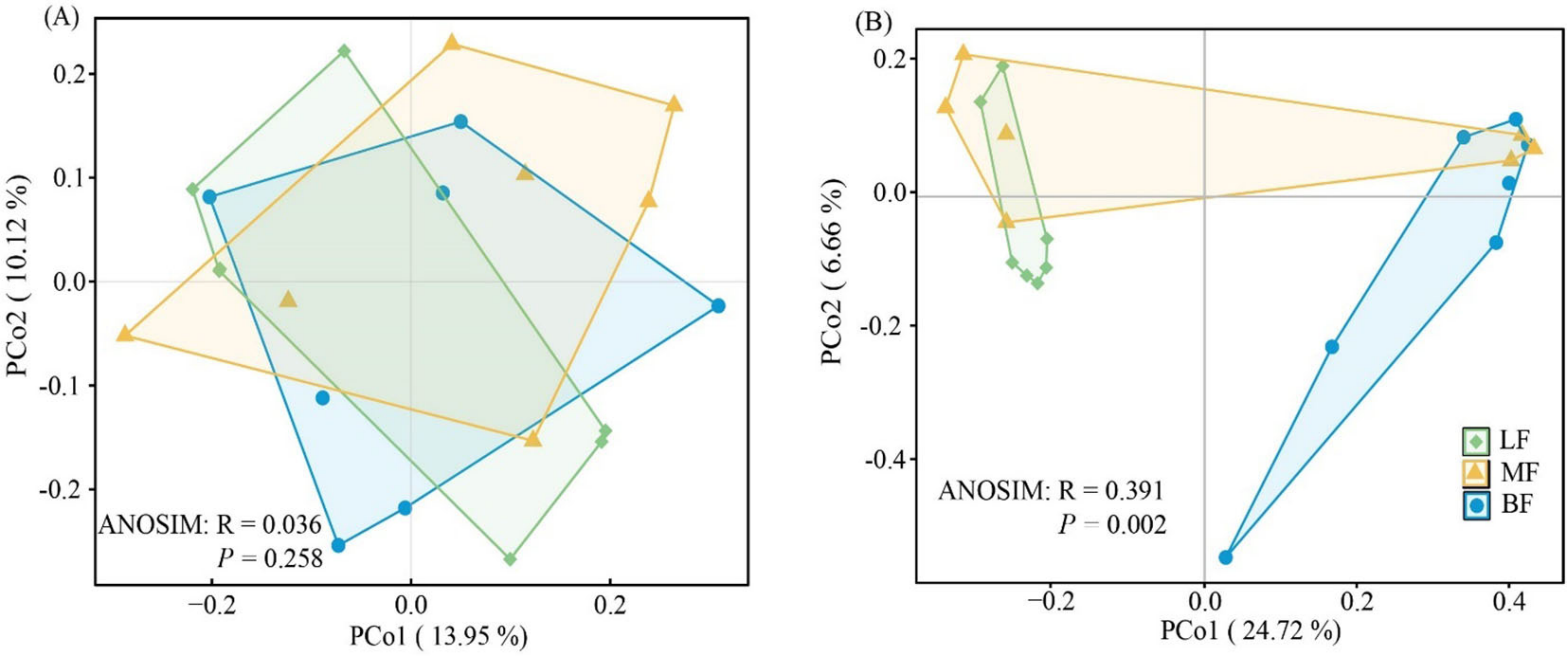
Principal coordinate analysis (PCoA) ordination and analysis of similarity (ANOSIM) based on the Bray-Curtis dissimilarity matrix of soil bacterial **(A)** and fungal **(B)** community composition across stand types.

PCoA based on Bray-Curtis distance was performed to assess the isolation of soil bacterial and fungal communities. The results showed no significant difference in bacterial community beta diversity among the three stand types, as confirmed by similarity analysis (ANOSIM, R = 0.036, *P =* 0.391, **Figure 3A**). In contrast, fungal communities from different stand types formed distinct clusters (ANOSIM, R = 0.391, *P* = 0.002, **Figure 3B**). The first two principal components of the PCoA explained 13.95% and 10.12% of the variation in bacterial communities, and 24.72% and 6.66% of the variation in fungal communities, respectively.

### 3.3 Co-occurrence networks of soil microorganisms

**Figure 4:**
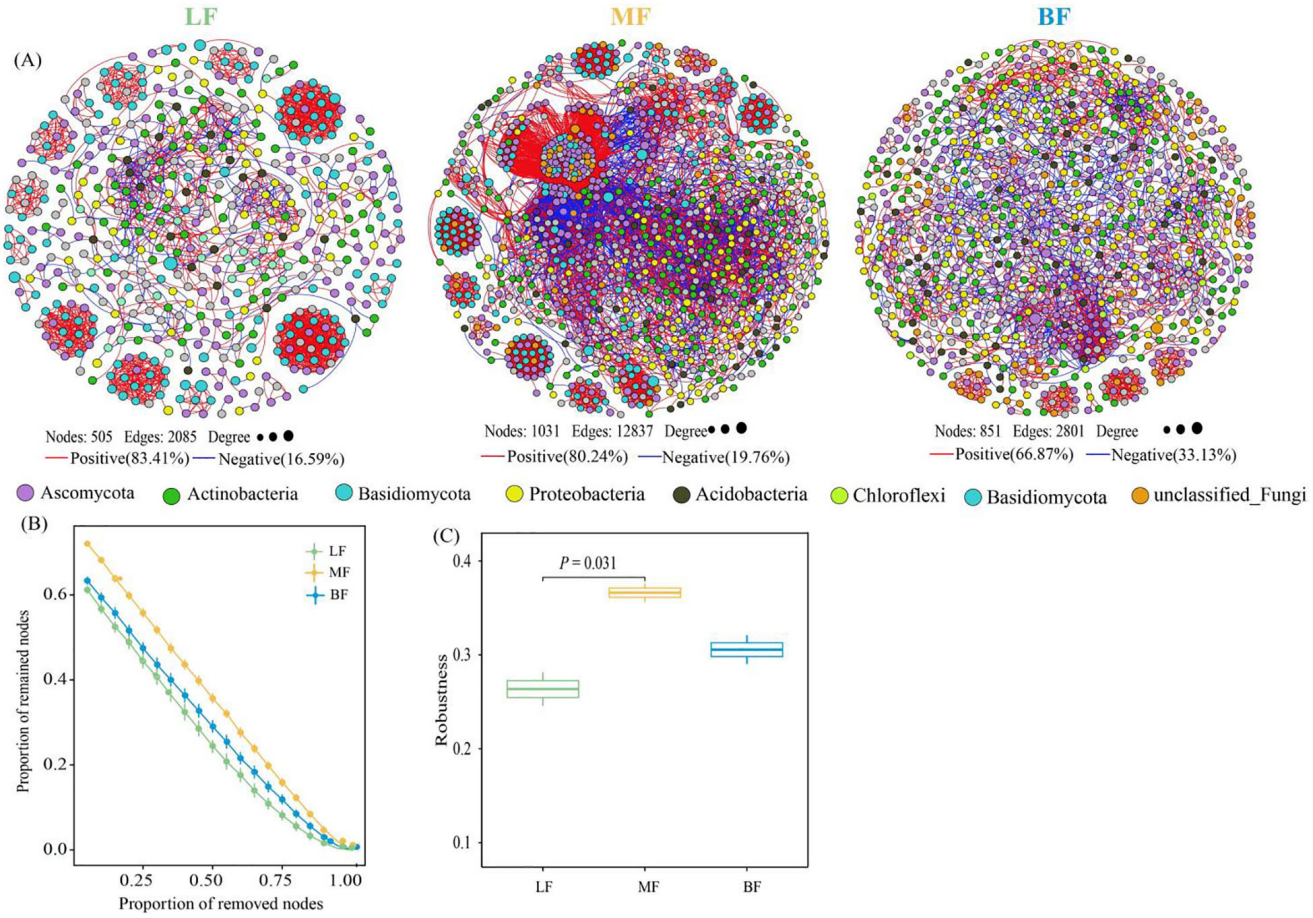
Co-occurrence network based on phylum-level microbial composition across different stand types **(A)**. Connections in the network represent strong (Spearman’s r absolute value > 0.7) and significant (*p*< 0.05) correlations. Node colors represent the microbial phylum to which each node belongs. The size of each node reflects its degree of connection. Red edges indicate positive interactions, while blue edges indicate negative interactions. The proportion of remaining nodes in the network after random node removal **(B)** and after randomly deleting 50% of the nodes **(C)** represents network stability.

To determine the impact of stand types on microbial network complexity, various topological parameters were extracted for networks from different stand types. The LF network consisted of 505 nodes and 2085 edges, the BF network had 851 nodes and 2801 edges, and the MF network comprised 1031 nodes and 12,837 edges. Compared with LF, both MF and BF networks exhibited higher average degrees. In LF co-occurrence networks, microbial interactions were primarily positive, whereas MF and BF networks showed an increased ratio of negative edges compared to LF. Furthermore, the MF network had a lower network diameter and average path length (13 and 4.439, respectively) compared to the other networks (**Figure 4A**; **Table 3**). Overall, network complexity decreased in the order of MF, BF, and LF. Based on random species loss or targeted removal of module hubs, the MF network demonstrated significantly higher robustness (*P<* 0.05) than LF (**Figures 4B, C**). In line with network complexity, network stability followed the order of MF, BF, and LF.

**Table 3:** Topological characteristics of microbial networks for different stand types.

Topological properties	Stand types		
	LF	MF	BF
Number of nodes	505	1031	851
Number of edges	2085	12837	2801
Negative edges (%)	16.590	19.76	33.130
Network diameter	22	13	19
Graph density	0.016	0.021	0.008
Average degree	6.583	13.807	8.257
Average path length	7.018	4.439	6.656
Average clustering coefficient	0.688	0.593	0.547
Modularity	0.873	0.615	0.845

### 3.4 Prediction of potential functions of soil microorganisms

**Figure 5:**
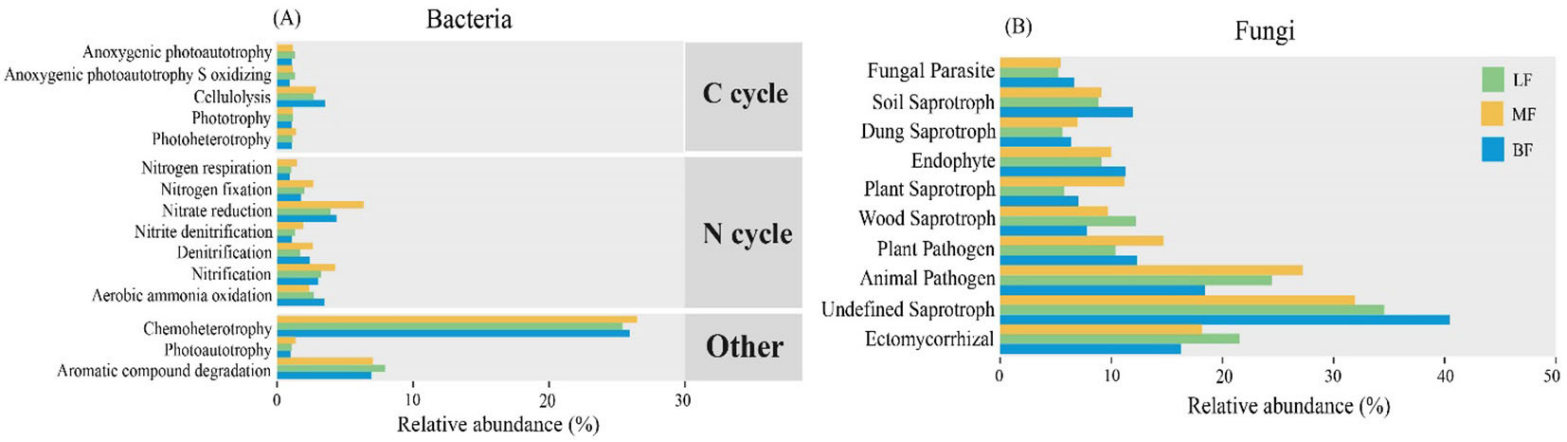
Variation in soil bacterial **(A)** and fungal **(B)** functional taxa across different stand types, based on FAPROTAX and FUNGuild databases, respectively.

Bacterial functional prediction was performed using the FAPROTAX database for annotation. As shown in **Figure 5**, the proportion of functional taxa involved in N cycling was higher in MF than in the other two stand types, including taxa related to nitrite respiration, nitrification, and denitrification. Furthermore, the overall abundance of chemoheterotrophic taxa was relatively high, especially in MF. The LF stand exhibited a higher abundance of taxa associated with aromatic compound degradation. In contrast, the relative abundance of cellulolysis-related functional taxa involved in carbon fixation was higher in BF. The FUNGuild database was used to predict the ecological functions of fungi, revealing a higher proportion of “saprotrophs” and “pathotrophs” compared to “symbiotrophs”. Furthermore, functional taxa associated with plant pathogens, plant saprotrophs, and animal pathogens were generally more abundance in MF. The functional taxa of ectomycorrhizal and wood saprotroph were more prevalent in LF, while BF exhibited higher abundances of soil saprotrophs, endophytes, and undefined saprotrophs.

### 3.5 Relationship between SQI, microbial diversity, network complexity, and keystone taxa

**Figure 6:**
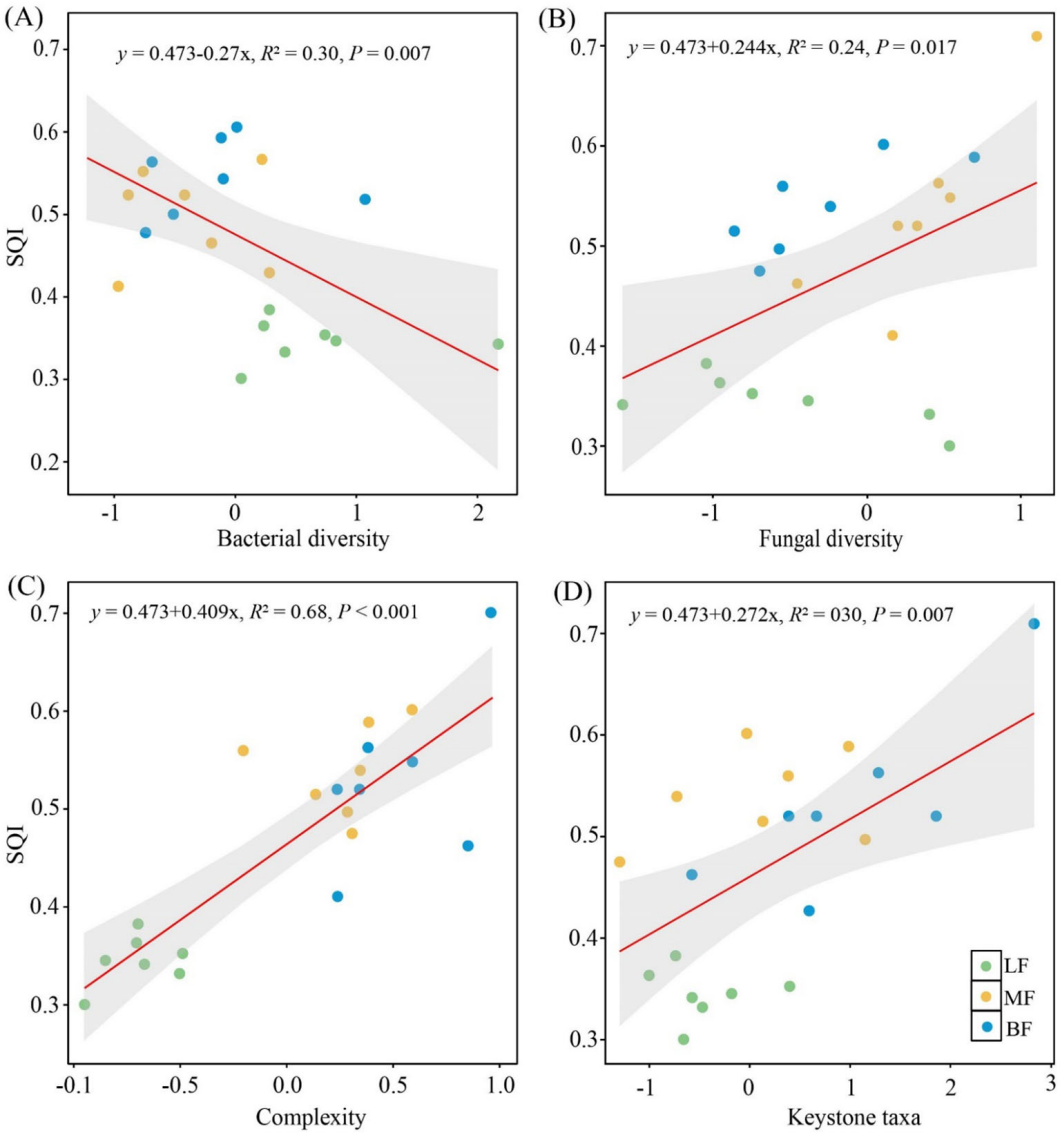
The relationship between microbial diversity **(A, B)**, network complexity **(C)**, relative abundance of keystone taxa **(D)**, and SQI. The shaded areas represent the 95% confidence interval.

Soil bacterial diversity (*R* ^2^ = 0.30, *P<* 0.05) was significantly negatively associated with soil quality (**Figure 6A**). However, both fungal diversity (*R* ^2^ = 0.24, *P<* 0.05) and network complexity (*R* ^2^ = 0.68, *P<* 0.001) were positively associated with soil quality (**Figures 6B, C**). Keystone taxa, which play key roles in shaping the network structure, were identified through the Zi-Pi plot (**Supplementary Figure S1**). A total of 21 module hubs (nodes with high connectivity to other members in a module) and 24 connectors (nodes linking different modules) were detected as keystone taxa. The number of keystone taxa was higher in MF (23) and BF (17) than in LF (5). At the phylum level, keystone bacterial taxa included Acidobacteria, Proteobacteria, Actinobacteria, Verrucomicrobia, and Chloroflexi, while fungal keystone taxa comprised Ascomycota, Mucoromycota, Basidiomycota, and Mortierellomycota (**Supplementary Table S4**). Additionally, soil quality was significantly related to the presence of keystone taxa (*R* ^2^ = 0.30, *P<* 0.05), with soil quality increasing in proportion to the relative abundance of keystone taxa (**Figure 6D**).

## 4 Discussion

### 4.1 Differences in soil characteristics across stand types

In the present study, significant differences in several soil properties were observed among stand types. The contents of SOC, TN, AN, and AP in MF were similar to that in BF, but higher than in LF (**Table 2**). This result is consistent with the findings of Li et al. (2023), indicating that mixed forests enhance nutrient availability and improve soil nutrient cycle (Liu et al., 2017; Likulunga et al., 2021). The observed effects can be attributed to the quality of plant litter (Thoms et al., 2010). Generally, coniferous forest litter has higher C/N ratios and lignin content. with a higher proportion of recalcitrant compounds compared to broadleaf forest litter (Cornwell et al., 2008). This characteristic leads to slower decomposition rates in larch forests, which may reduce soil fertility (Ge et al., 2023). In contrast, the MF increases litter diversity and accelerates the accumulation of soil humus and other nutrients, thereby promoting soil fertility (Mueller et al., 2015; Wang et al., 2021). Additionally, bulk density (BD) was higher in LF stands and relatively lower in BF and MF stands (**Table 2**). The lower BD, looser structure, and increased pore space in MF stands contribute to stronger water and nutrient retention capacity (Xiang et al., 2023). In this study, the pH was higher in BF stands (**Table 2**), likely due to the higher exchangeable cations, such as Mg^2+^ and Ca^2+^, found in broadleaf forest litter, which enhance soil buffering capacity and raise pH (Tóth et al., 2011). In summary, compared to coniferous forests, mixed coniferous and broad forests exhibited higher nutrient availability.

### 4.2 Changes in microbial community characteristics across stand types

Forest stand types are likely to influence soil microbial community characteristics indirectly by altering soil physicochemical properties. Although the Chao1 index of bacteria and fungi showed no significant differences among stand types, significant differences were found in the Shannon index (**Figures 2A, C**). Specifically, the bacterial Shannon index was lower in MF and BF stands, while the fungal Shannon index was higher in these stands. Soil factors were closely related to the diversity of both soil bacteria and fungi (Yarwood and Hogberg, 2017). Bacterial diversity exhibited a negative correlation with SOC, TN, TP, AN, and AP, while fungal diversity showed a significant positive correlation (**Supplementary Figure S3**). Soil nutrient content was higher in MF and BF stands compared to LF stands, with elevated levels of SOC, TN, TP, AN, and AP (**Table 2**). Consequently, MF and BF stands likely provide favorable microhabitats for fungal activity and growth by enhancing soil nutrient availability, which in turn increases fungal diversity. In this study, bacterial community composition did not differ significantly across stand types, but the fungal community was distinctly separated by stand type. Ascomycota contribute to the decomposition of litter and organic matter and are generally considered cellulolytic, with limited lignin-decomposing ability (Osono, 2007). The increase in litter diversity observed in MF stands, which is associated with higher levels of SOC and other nutrients (**Table 2**), may explain the increased relative abundance of Ascomycota in MF stands. The abundance of Ascomycota in BF stands was similarly high, potentially due to the elevated pH levels (**Figure 2F**; **Table 2**). Previous studies have shown that higher pH levels correlate with greater Ascomycota abundance (Hou et al., 2024). In contrast, Basidiomycota are more strongly correlated with litter biomass and are well-known for their ability to decompose lignin, a component of litter that is otherwise difficult to break down (Lundell et al., 2010). Given that conifer litter is more challenging to decompose than broadleaf litter (Chen et al., 2019a), non-degradable carbon accumulates more readily in LF stands, leading to a higher abundance of Basidiomycota in these stands.

### 4.3 Differences in co-occurrence networks and keystone taxa across stand types

Differences in microbial co-occurrence network structure, including complexity and stability, were observed among the three stand types. The soil microbial network in MF stands was found to be more complex and stable (**Figure 4A**), consistent with the findings of Xu et al. (2023). Additionally, the MF stands had a relatively high ratio of negative edges in bacterial taxa networks, whereas the LF stands displayed a greater ratio of positive edges in fungal taxa networks (**Table 3**). This pattern suggests that tree species mixing in MF stands increases potential competition among bacterial taxa, while LF stands facilitate greater potential cooperation among fungal taxa. As highlighted in previous studies, the structure of soil microbial communities is susceptible to environmental factors, especially changes in soil physicochemical properties (Nkongolo and Narendrula-Kotha, 2020; Zhang et al., 2023). Specifically, the contents of SOC, TN, TP, BD, and pH mainly affected the bacterial community (**Supplementary Figure S4A**), whereas SOC, TN, AN, MWHC, and pH were the key factors (*P*< 0.01) influencing the fungal community (**Supplementary Figure S4B**). Consequently, stand types can alter the composition of keystone microbial taxa (Li et al., 2023). More keystone taxa were identified in MF and BF networks compared to the LF network, with most taxa belonging to *Proteobacteria*, *Actinobacteria*, *Verrucomicrobia, Ascomycota*, *Basidiomycota*, or *Mortierellomycota*. Furthermore, more keystone fungal taxa than bacterial taxa were observed (**Supplementary Figure S1**; **Supplementary Table S4**). These keystone taxa, identified based on their topological roles as module hubs and connectors, play central roles in soil biogeochemical processes by mediating inter-module substrate exchange and maintaining network stability (Banerjee et al., 2018; Li et al., 2023). Their removal has been to disrupt both the structure and function of soil microbiomes (Berry and Widder, 2014). Many of these keystone taxa are associated with soil nutrient cycling and transformation. For example, keystone connectors such as the *Mesorhizobium* genus have been found to enhance soil nitrogen availability by linking nitrogen-fixing modules with root-associated networks (Zhong et al., 2019; Laranjo et al., 2014). Meanwhile, module hubs like the *Saitozyma* genus contribute to organic matter decomposition by coordinating lignin-degrading modules (Lundell et al., 2010).

### 4.4 Effects of stand type and microbial community on SQI

It was observed that stand type influenced SQI, with SQI being generally higher in MF and BF compared to LF (**Table 2**). The superior soil properties of MF stands were the primary reason for their highest SQI. Consistent with prior findings, soil with relatively high levels of SOC, TN, AN, and lower BD tend to exhibit improved soil quality (Shao et al., 2020). Plants contribute carbon and energy to the soil through root secretions and litter. In stands with mixed birch and larch, increased tree species diversity and litter return enhance soil quality by providing organic matter inputs and improving nutrient availability (Bu et al., 2019). Microorganisms, as key drivers of soil ecological restoration, are influenced by resource allocation strategies and regulated by shifts in community metabolism (Chen et al., 2022). Thus, stand type can indirectly alter soil microbial communities, thereby affecting soil quality. It was demonstrated that the bacterial Shannon index was lower in MF stands, while the fungal Shannon index was higher. Further analyses indicated that the SQI exhibited a strong negative relationship with soil bacterial diversity (**Figure 6A**), whereas a significant positive association was observed with the increase in fungal diversity (**Figure 6B**), consistent with previous findings (Wan et al., 2024). This pattern may be attributed to the fact that higher bacterial diversity can reduce interspecies cooperation and intensify competition for energy and nutrients (Palmer and Foster, 2022), ultimately exerting a negative impact on soil quality. In contrast, the positive association between fungal diversity and soil quality is likely due to the key roles of fungi in decomposing soil organic matter (Zhong et al., 2018; Yan et al., 2020). Specifically, saprophytic fungi are particularly important in breaking down complex organic materials (Wan et al., 2024), facilitating the release of nutrients. Additionally, many fungi form biotrophic relationships with plant roots (Zhong et al., 2018; Liu et al., 2024), enhancing nutrients absorption by plants and improving soil quality through increased plant litter input (Wan et al., 2024). Moreover, soil nutrient availability and substrate quality are known to influence specific microbial taxa (Xu et al., 2021). In this study, a greater number of keystone taxa were identified in the MF microbial network, and their relative abundance showed a positive relationship with SQI (**Figure 6D**). Soil quality was significantly related to these keystone taxa through their role in shaping the structure and function of microbial co-occurrence networks (Berry and Widder, 2014; Banerjee et al., 2018). Additionally, it was observed that soil quality improved as microbial network complexity increased (**Figure 6C**).

We used FAPROTAX and FUNGuild software to further predict the ecological functions of bacterial and fungal taxa in different forest stand types. The relative abundance of bacterial taxa associated with nitrite respiration, nitrification, denitrification, and chemoheterotroph was higher in MF stands (**Figure 5A**). These bacterial taxa play essential roles in the cycling of organic matter and nitrogen in ecosystems (Gu et al., 2019), enhancing nutrient availability in MF stands (**Table 2**) and alleviating resource limitations for certain microbial taxes. Regarding fungal taxa, the proportions of “saprotroph” and “pathotroph” were higher in MF and BF stands, whereas ectomycorrhizal and wood saprotroph taxa were abundant in LF stands (**Figure 5B**). This distribution may be attributed to differences in soil nutrient availability, litter quality, tree species characteristics, and the colonization strategies of fungal communities (Xu et al., 2021). As an ectomycorrhizal species, larch produces litter with high lignin content (Cornwell et al., 2008), which promotes the colonization of decomposer communities adapted to lower nutrient conditions (Asplund et al., 2019). In conclusion, stand types could influence microbial network complexity and stability by regulating keystone taxa, thereby affecting soil quality and ecosystem functions.

## 5 Conclusion

This study revealed the mechanisms driving SQI improvement across different stand types from a microbiological perspective. It was demonstrated that MF stands reduced soil physical properties (BD), improved nutrient availability (SOC, TN, AN, and AP), increased soil fungal diversity, and promoted greater microbial network complexity and stability. Furthermore, the relative abundance of keystone bacterial and fungal taxa involved in soil carbon and nitrogen cycling was shown to contribute to soil quality improvement. In summary, stand types influence soil quality both directly, by altering soil properties, and indirectly, by affecting microbial diversity, network complexity and stability, as well as the abundance of keystone taxa. These findings offer valuable insights into soil nutrient cycling mechanisms and provide a basis for improving forest management practices. For instance, promoting symbiotic relationships between plants and fungi by selecting suitable plant species or optimizing mixed forest ratios can improve soil structure and nutrient cycling. Additionally, introducing keystone saprophytic fungi microbial groups are proposed to improve soil quality by accelerating the decomposition of soil organic matter and facilitating nutrient release.

## Data Availability

The original contributions presented in the study are included in the article/**Supplementary Material**. Further inquiries can be directed to the corresponding author.
