# Supplementary material for: Microbial determinants of soil quality in mixed larch and birch forests: network structure and keystone taxa abundances
Source: Front Plant Sci. 2025 Jul 15;16:1491038. doi: 10.3389/fpls.2025.1491038 (PMC12303966; doi:10.3389/fpls.2025.1491038)
Supplement: Supplementary file 1 [file DataSheet1.docx]

Supplementary Material

**Supplementary Table 1.** Results of principal component analysis (PCA) of soil quality indicators for three stand types. Bolded values indicate highly weighted load factors, while underlined values are factors retained in the minimum data set (MDS).

| Principal components | PC-1 | PC-2 | PC-3 |
| --- | --- | --- | --- |
| Eigenvalues | 4.92 | 1.39 | 1.08 |
| Variance (%) | 0.62 | 0.17 | 0.14 |
| Cumulative (%) | 0.60 | 0.77 | 0.93 |
| Weighting value | 0.66 | 0.19 | 0.15 |
| SOC | **-0.425** | -0.080 | -0.099 |
| TN | **-0.420** | -0.032 | -0.244 |
| TP | -0.308 | -0.420 | -0.313 |
| AN | **-0.438** | -0.021 | -0.118 |
| AP | -0.344 | 0.377 | -0.324 |
| pH | -0.064 | **0.819** | -0.047 |
| MWHC | -0.353 | -0.025 | 0.561 |
| BD | 0.33 | -0.04 | **-0.63** |

**Supplementary Table 2.** Non-linear weighted scores for indicators.

| Parameter | Average | Slop (b) | Normalization equation | Weighting value (*W*) |
| --- | --- | --- | --- | --- |
|  | (x_0_) |  |  |  |
| AN | 468.05 | -2.5 | *S* = 1/(1+(x/468.05)^-2.5^) | 0.66 |
| pH | 6.21 | 2.5 | *S* = 1/(1+(x/6.21)^2.5^) | 0.19 |
| BD | 0.87 | 2.5 | *S* = 1/(1+(x/0.87)^2.5^) | 0.15 |

**Supplementary Table 3.** The Pearson correlation analysis of soil physicochemical indicators in three stand types. ** P < 0.01; * P < 0.05.

|  | SOC | TN | TP | AN | AP | PH | MWHC | BD |
| --- | --- | --- | --- | --- | --- | --- | --- | --- |
| SOC | 1 |  |  |  |  |  |  |  |
| TN | 0.977 | 1 |  |  |  |  |  |  |
| TP | 0.473 | 0.491 | 1 |  |  |  |  |  |
| AN | 0.955 | 0.951 | 0.502 | 1 |  |  |  |  |
| AP | 0.733 | 0.751 | 0.293 | 0.732 | 1 |  |  |  |
| pH | -0.163 | -0.098 | -0.241 | -0.129 | 0.280 | 1 |  |  |
| MWHC | 0.511 | 0.402 | 0.166 | 0.517 | 0.322 | -0.098 | 1 | -0.947 |
| BD | -0.506 | -0.411 | 0.001 | -0.475 | -0.315 | 0.099 | -0.947 | 1 |

**Supplementary Table 4.** List of keystone taxa of bacteria and fungi in different stand types.

| Stand  types | ASV  number | Taxonomy | Role | Phylum | Genus | Relative  abundance (%) |
| --- | --- | --- | --- | --- | --- | --- |
| LF | ASV_67 | Bacteria | Module hubs | Acidobacteria | *RB41* | 0.035 |
|  | ASV_129 | Bacteria | Module hubs | Proteobacteria | *uncultured* | 0.042 |
|  | ASV_88 | Bacteria | Connectors | Proteobacteria | *Dongia* | 0.060 |
|  | ASV_92 | Bacteria | Connectors | Proteobacteria | *Mesorhizobium* | 0.045 |
|  | ASV_ 93085 | Fungi | Connectors | Ascomycota | *Tetracladium* | 0.031 |
| MF | ASV_14 | Bacteria | Module hubs | Actinobacteria | *MB-A2-108* | 0.134 |
|  | ASV_31 | Bacteria | Module hubs | Actinobacteria | *Mycobacterium* | 0.064 |
|  | ASV_145 | Bacteria | Module hubs | Actinobacteria | *unclassified_Micrococcaceae* | 0.043 |
|  | ASV_320 | Bacteria | Module hubs | Verrucomicrobia | *Candidatus_Udaeobacter* | 0.016 |
|  | ASV_512 | Bacteria | Module hubs | Actinobacteria | *Nocardioides* | 0.012 |
|  | ASV_579 | Bacteria | Module hubs | Chloroflexi | *TK10* | 0.015 |
|  | ASV_92776 | Fungi | Module hubs | Ascomycota | *unclassified_Pezizales* | 0.098 |
|  | ASV_92991 | Fungi | Module hubs | Mucoromycota | *Umbelopsis* | 0.035 |
|  | ASV_93192 | Fungi | Module hubs | Basidiomycota | *Saitozyma* | 0.018 |
|  | ASV_93763 | Fungi | Module hubs | Ascomycota | *Chaetomium* | 0.010 |
|  | ASV_438 | Bacteria | Connectors | Actinobacteria | *Luedemannella* | 0.015 |
|  | ASV_521 | Bacteria | Connectors | Acidobacteria | *Subgroup_6* | 0.012 |
|  | ASV_851 | Bacteria | Connectors | Proteobacteria | *bacteriap25* | 0.011 |
|  | ASV_92673 | Fungi | Connectors | Basidiomycota | *Solicoccozyma* | 0.448 |
|  | ASV_92694 | Fungi | Connectors | unclassified_Fungi | *unclassified_Fungi* | 0.411 |
|  | ASV_92715 | Fungi | Connectors | Mortierellomycota | *Mortierella* | 0.203 |
|  | ASV_92723 | Fungi | Connectors | Mortierellomycota | *Mortierella* | 0.107 |
|  | ASV_92767 | Fungi | Connectors | Basidiomycota | *Inocybe* | 0.387 |
|  | ASV_92792 | Fungi | Connectors | Basidiomycota | *Solicoccozyma* | 0.168 |
|  | ASV_92797 | Fungi | Connectors | Ascomycota | *unclassified_Chaetothyriales* | 0.093 |
|  | ASV_92810 | Fungi | Connectors | Ascomycota | *Knufia* | 0.067 |
|  | ASV_92873 | Fungi | Connectors | Ascomycota | *unclassified_Helotiales* | 0.062 |
|  | ASV_92949 | Fungi | Connectors | unidentified | *unidentified* | 0.038 |
| BF | ASV_156 | Bacteria | Module hubs | Verrucomicrobia | *Candidatus_Udaeobacter* | 0.027 |
|  | ASV_276 | Bacteria | Module hubs | Actinobacteria | *MB-A2-108* | 0.012 |
|  | ASV_299 | Bacteria | Module hubs | Proteobacteria | *unclassified_Methyloligellaceae* | 0.014 |
|  | ASV_480 | Bacteria | Module hubs | Proteobacteria | *TRA3-20* | 0.017 |
|  | ASV_511 | Bacteria | Module hubs | Proteobacteria | *SC-I-84* | 0.012 |
|  | ASV_92919 | Fungi | Module hubs | Ascomycota | *unidentified* | 0.068 |
|  | ASV_93096 | Fungi | Module hubs | Mortierellomycota | *Mortierella* | 0.077 |
|  | ASV_93334 | Fungi | Module hubs | Ascomycota | *Stachybotrys* | 0.024 |
|  | ASV_93396 | Fungi | Module hubs | Ascomycota | *Ophiosphaerella* | 0.033 |
|  | ASV_219 | Bacteria | Connectors | Actinobacteria | *Mycobacterium* | 0.015 |
|  | ASV_417 | Bacteria | Connectors | Actinobacteria | *uncultured* | 0.010 |
|  | ASV_657 | Bacteria | Connectors | Actinobacteria | *Actinoplanes* | 0.013 |
|  | ASV_962 | Bacteria | Connectors | Actinobacteria | *67-14* | 0.011 |
|  | ASV_92653 | Fungi | Connectors | Ascomycota | *Myrmecridium* | 1.228 |
|  | ASV_92851 | Fungi | Connectors | Ascomycota | *unidentified* | 0.176 |
|  | ASV_92994 | Fungi | Connectors | Ascomycota | *unidentified* | 0.073 |
|  | ASV_93278 | Fungi | Connectors | Ascomycota | *Coniochaeta* | 0.019 |


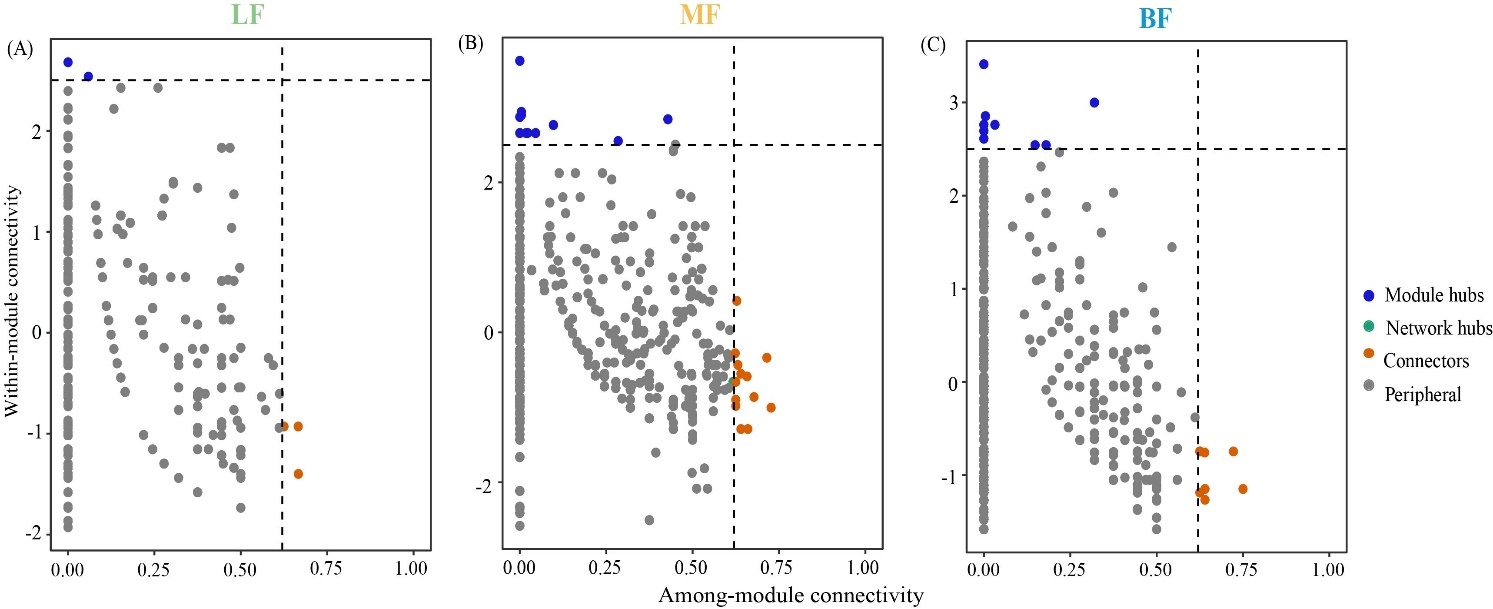


**Figure S1** The Zi-Pi score for topological roles of each node in Figure 5.


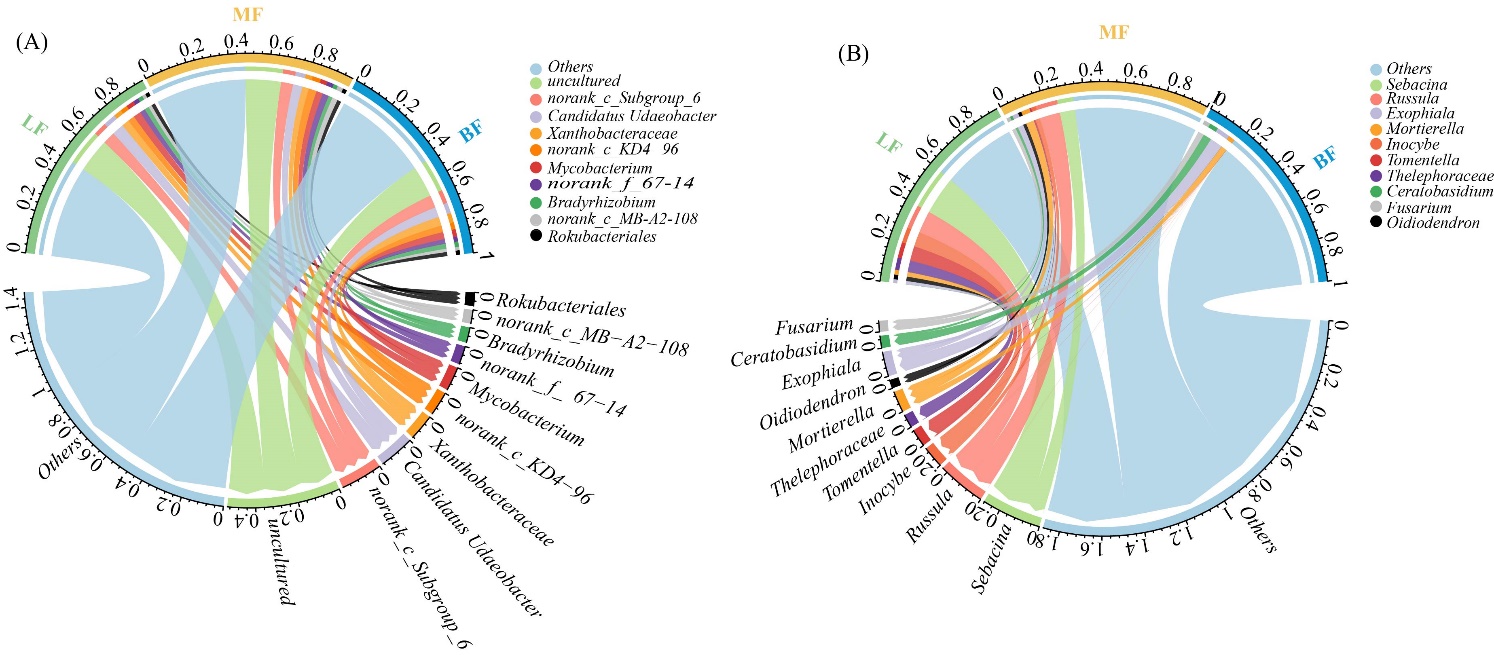


**Figure S2** Circos plots showing the taxonomic composition of soil bacterial (A) and fungal (B) taxa of different stand types at the genus level.


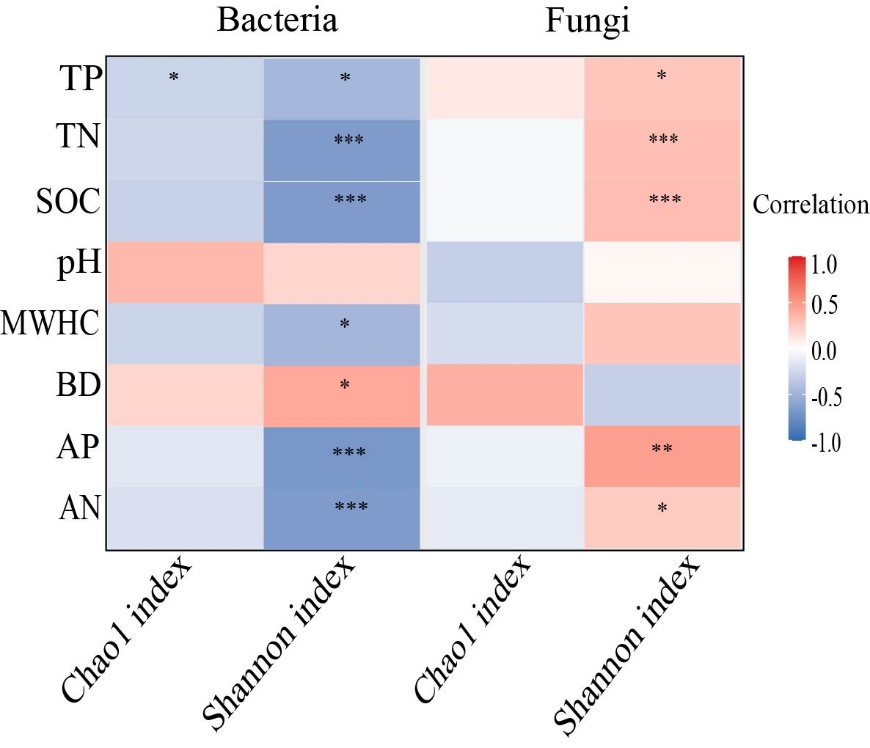


**Figure S3** Spearman's correlation analysis between soil physicochemical characteristics and bacterial **(A)** and fungal **(B)** alpha diversity index. Colors represent the correlations, with red indicating positive correlations and blue indicating negative correlations. *** *P* < 0.001; ** *P* < 0.01; * *P* < 0.05.


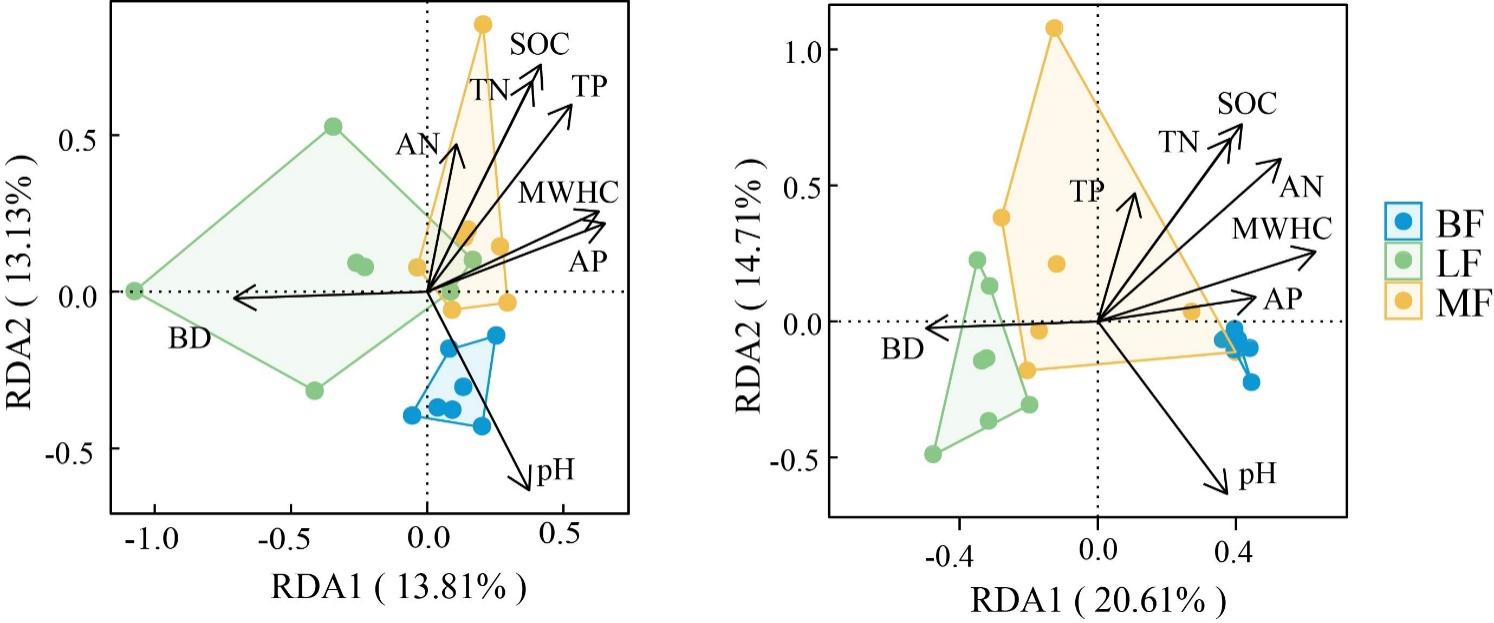


**Figure S4** The relationship between bacterial (A) and fungal (B) community structure and soil properties was evaluated by redundancy analysis. The percentage in parentheses represents the variation explained by each axis.
